# *In vivo* functional dissection of a context-dependent role for Hif1α in pancreatic tumorigenesis

**DOI:** 10.1038/oncsis.2016.78

**Published:** 2016-12-12

**Authors:** T Cheng, Z Jian, K Li, S Raulefs, I Regel, S Shen, X Zou, J Ruland, G O Ceyhan, H Friess, C W Michalski, J Kleeff, B Kong

**Affiliations:** 1 Department of Surgery, Technical University of Munich (TUM), Munich, Germany; 2 Institute of Clinical Chemistry and Pathobiochemistry, Munich, Germany; 3 Institute of Pathology, Heinrich-Heine-University, Duesseldorf, Germany; 4 Department of Gastroenterology, the Affiliated Drum Tower Hospital of Nanjing University, Medical School, Nanjing, Jiangsu, China; 5 German Cancer Consortium (DKTK), Heidelberg, Germany; 6 German Center for Infection Research (DZIF), Munich, Germany; 7 Department of Surgery, University of Heidelberg, Heidelberg, Germany; 8 NIHR Pancreas Biomedical Research Unit, Department of Molecular and Clinical Cancer Medicine, University of Liverpool, Liverpool, UK

## Abstract

Hypoxia-inducible factor 1α (Hif1α) is a key regulator of cellular adaptation and survival under hypoxic conditions. In pancreatic ductal adenocarcinoma (PDAC), it has been recently shown that genetic ablation of Hif1α accelerates tumour development by promoting tumour-supportive inflammation in mice, questioning its role as the key downstream target of many oncogenic signals of PDAC. Likely, Hif1α has a context-dependent role in pancreatic tumorigenesis. To further analyse this, murine PDAC cell lines with reduced Hif1α expression were generated using shRNA transfection. Cells were transplanted into wild-type mice through orthotopic or portal vein injection in order to test the *in vivo* function of Hif1α in two major tumour-associated biological scenarios: primary tumour growth and remote colonization/metastasis. Although Hif1α protects PDAC cells from stress-induced cell deaths in both scenarios—in line with the general function Hif1α—its depletion leads to different oncogenic consequences. Hif1α depletion results in rapid tumour growth with marked hypoxia-induced cell death, which potentially leads to a persistent tumour-sustaining inflammatory response. However, it simultaneously reduces tumour colonization and hepatic metastases by increasing the susceptibility to anoikis induced by anchorage-independent conditions. Taken together, the role of Hif1α in pancreatic tumorigenesis is context-dependent. Clinical trials of Hif1α inhibitors need to take this into account, targeting the appropriate scenario, for example palliative vs adjuvant therapy.

## Introduction

Pancreatic ductal adenocarcinoma (PDAC) is a devastating cancer entity characterized by tissue hypoxia.^1, 2^ Activated by hypoxia, hypoxia-inducible factor 1α (Hif1α) is a key downstream effector, which is involved in a variety of cellular processes mediating a number of adaptive changes. These adaptive changes are meant to alleviate cellular stress induced by hypoxic conditions and to promote cell survival under physiological circumstances.^3, 4^ However, these responses are frequently used by PDAC cells to enhance the malignant potential such as chemoresistance or metastatic invasion.^5, 6^ Indeed, high expression of Hif1α is a negative predictor of PDAC patient's overall survival.^7^ Besides, under normoxic conditions, Hif1α acts as the key downstream target of a number of putative oncogenic pathways of PDAC for example mTOR (mechanistic target of rapamycin). Hyperactivated mTOR signalling is able to stabilize Hif1α in the absence of hypoxia and promotes tumour angiogenesis.^8^ Collectively, these data argue for an oncogenic role of Hif1α in pancreatic tumorigenesis.

Recently, this notion has been challenged by a provocative study showing that genetic ablation of Hif1α significantly accelerated PDAC development in mice by promoting B lymphocyte-mediated tumour-supportive inflammation.^9^ The crucial crosstalk with the immune system points to a complex function of Hif1α in pancreatic tumorigenesis. Thus, we hypothesised that Hif1α might have a context-dependent role in PDAC. To test this, we generated murine PDAC cell lines with reduced Hif1α expression from previously established cell lines.^10^ The *in vivo* function of Hif1α was tested in two major tumour-associated biological scenarios: primary tumour growth and remote colonization/metastatic growth.

## Results and Discussion

**Figure 1:**
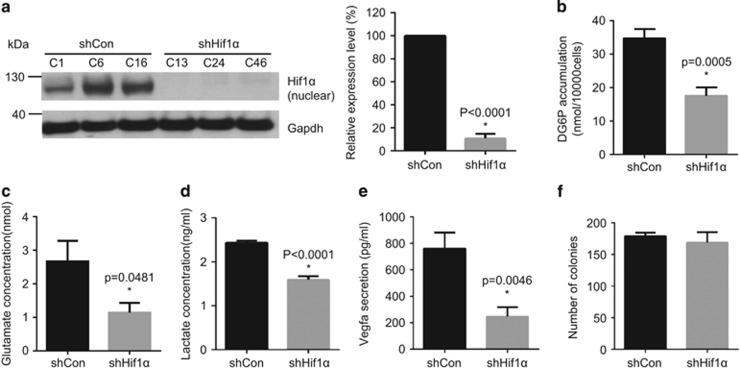
Hif1α is downregulated in murine PDAC cells. (**a**) Western blot analyses show the downregulation of Hif1α in shHif1α cells but not in shControl cells: (left) western blot result; and (right) quantitative measurement; shHif1α: transfection with Hif1α-specific shRNA-expressing vector; shControl: transfection with control vectors. (**b–d**) The metabolic state of shControl cells or shHif1α cells is determined by glucose uptake assay (**b**), glutamate assay (**c**) and lactate secretion assay (**d**). (**e**) Vegfa secretion between shControl and shHif1α cells is measured by Vegfa ELISA assay. (**f**) The proliferation capacity of shControl or shHif1α cells is determined by colony formation assay. All data are presented as mean±s.e.m. (*n*=3), unpaired *t*-test is used to examine statistical significance, *P<0.05. See Supplementary Materials and Methods.

Previously, we characterized a number of murine PDAC cell lines from *p48*^*Cre*^*; Kras*^G12D/+^*; Tsc1*^flox/+^mice.^1, 10^ Among these, 399 cells characterized by hyperactivated Mek/Erk/mTOR signalling reliably develop tumours in wild-type mice (C57BL/6J) upon orthotopic or portal vein injection, and were therefore chosen for this study. We stably transfected 399 cells with scramble control (shControl) or Hif1α-specific shRNA-expressing plasmids (shHif1α). Western blot analysis confirmed an approximate 90% reduction in Hif1α expression in shHif1α cells compared with shControl cells (Figure 1a). In comparison with the control cells, shHif1α cells showed a significant reduction in glucose uptake (quantified by intracellular DG6P level: 50% reduction, *P*=0.0005) (Figure 1b), intercellular glutamate level (57% decrease, *P*=0.0481, Figure 1c) and lactate secretion (34% reduction, *P*<0.0001, Figure 1d), which is in line with the influence of Hif1α on tumour metabolism. Secretion of Vegfa (vascular endothelial growth factor A)—a downstream target of Hif1α—was also decreased in shHif1α cells (ELISA, 67% reduction, *P*=0.0046 Figure 1e). Downregulation of Hif1α had no effect on cell proliferation *in vitro* under anchorage-dependent conditions in the colony formation assay (Figure 1f).

**Figure 2:**
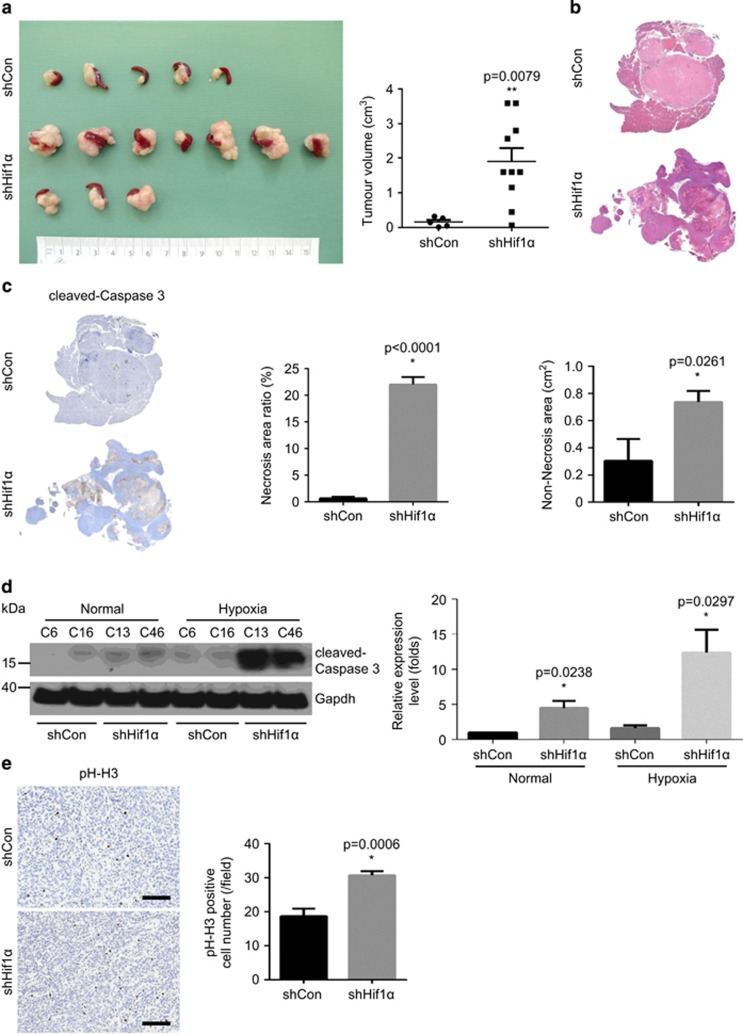
Hif1α depletion promotes primary tumour growth with tissue necrosis *in vivo*. (**a**) The orthotopic transplantation experiment shows the volume of tumours derived from shControl and shHif1α cells-injected animals: (left) gross pathology; and (right) quantitative measurement. (**b**) Representative H&E staining pictures show larger necrotic regions in shHif1α tumours compared with shControl tumours. (**c**) Immunohistochemistry (IHC) stainings of cleaved-caspase 3 (left) demonstrate more apoptotic cells (middle) and larger non-necrosis area (right) in shHif1α tumours in comparison with shControl tumours. (**d**) Western blot (left) and quantification results (right) show increased expression of cleaved-caspase 3 in shHif1α cells under hypoxic conditions. (**e**) IHC staining of phosph-histone H3 (pH-H3) and quantitative analysis demonstrates increased proliferation in shHif1α tumours; scale bar, 100 μm. See Supplementary Materials and Methods.

To test the influence of Hif1α depletion on primary tumour growth, we performed orthotopic injections using control and shHif1α cells. Interestingly, the shHif1α cells gave rise to significantly larger tumours compared with control cells (Figure 2a, 11.8-fold increase, *P*=0.0079). Histological analyse revealed that the tumours derived from shHif1α cells were more necrotic (Figure 2b). Indeed, the quantitative analyse confirmed that the necrotic region (boundary labelled by cleaved-caspase 3) was more pronounced in shHif1α cells-derived tumours (Figure 2c, 33.8-fold increase, *P*<0.0001). In line, shHif1α cells were more vulnerable to hypoxia-induced cell death *in vitro* (Figure 2d, 7.8-fold increase, *P*=0.0297). Since the shHif1α cells-derived tumours were larger even after excluding necrotic areas (Figure 2c, shCon vs shHif1α: 0.3±0.16 vs 0.7±0.08 cm^2^, *P*=0.0081), we hypothesized that the outgrowth of shHif1α tumours was due to elevated proliferation. Indeed, the quantitative analysis revealed that the proliferation index in viable tumour regions was significantly higher in shHif1α tumours compared with control tumours (1.6-fold change, *P*=0.0006) (Figure 2e).

**Figure 3:**
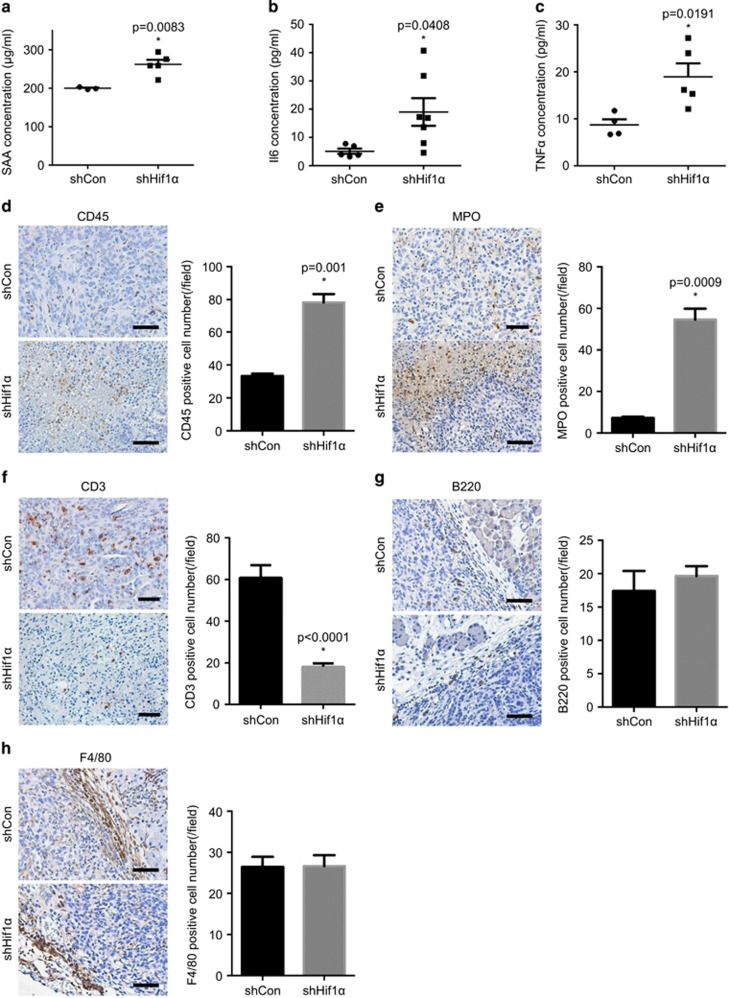
Increased inflammatory response triggered by Hif1α depletion. (**a–c**) Elevated levels of SAA (**a**), Il6 (**b**) and TNFα (**c**) are detected in the serums of shHif1α cells-transplanted animals compared with controls. (**d**) Representative IHC pictures of CD45 and quantitative analysis demonstrate increased immune cells infiltration (especially in necrotic areas) in the shHif1α tumours. (**e**) Representative IHC pictures of MPO and quantitative analysis reveal that neutrophils are the most infiltrated immune cells in the shHif1α tumours. (**f**) IHC staining of CD3 and quantitative analysis show reduced T-cell infiltration in the shHif1α tumours, which is confirmed by the quantitative analysis. Scale bar, 200 μm. (**g–h**) Representative IHC pictures and quantitative analysis of B220 (**g**) and F4/80 (**h**) show no difference in B cells and macrophages infiltration between shControl and shHif1α tumours. Scale bar, 200 μm. All data are presented as mean±s.e.m., and the statistical difference is determined by unpaired *t*-test. *P<0.05. See Supplementary Materials and Methods.

Since tumour-associated inflammation has been proven to affect tumour proliferation under certain conditions,^11^ we hypothesized that the outgrowth of shHif1α tumours resulted in necrosis that triggered an increased inflammatory response, which further supported rapid tumour growth. To test this, we measured the serum level of a number of inflammatory markers including serum amyloid A (SAA), Il6 (interleukin 6) and TNFα (tumour necrosis factor α). SAA levels in the serum of shHif1α cells-transplanted animals were significantly higher than controls (262.0±12.01 vs 199.8±1.92 μg/ml, *P*=0.0083) (Figure 3a). Similar results were obtained when serum levels of Il6 (Figure 3b, 19.0±4.89 vs 5.1±0.94 pg/ml, *P*=0.0408) and TNFα (Figure 3c, 19.0±2.84 vs 8.7±1.20 pg/ml, *P*=0.0191) were measured. Next, we profiled the immune cell infiltration in shHif1α and control cells-derived tumours by staining a number of immune cell markers including CD45 (immune cells), MPO (myeloperoxidase, neutrophils), CD3 (T cells), B220 (B cells) and F4/80 (macrophages). This analyses revealed that shHif1α tumours were more densely infiltrated by immune cells (especially in necrotic areas) than control tumours (2.4-fold change, *P*=0.001, Figure 3d). Subsequent analysis revealed that these immune cells mainly were neutrophils (Figure 3e, 7.6-fold change, *P*=0.0009). T-cell infiltration, however, was reduced in shHif1α tumours (Figure 3f). No difference in the number of infiltrating B cells (Figure 3g) and macrophages (Figure 3h) was observed. Taken together, the loss of Hif1α promoted rapid primary tumour growth, resulting in necrosis and triggering tissue necrosis-associated inflammation, which potentially further facilitated tumour growth.

**Figure 4:**
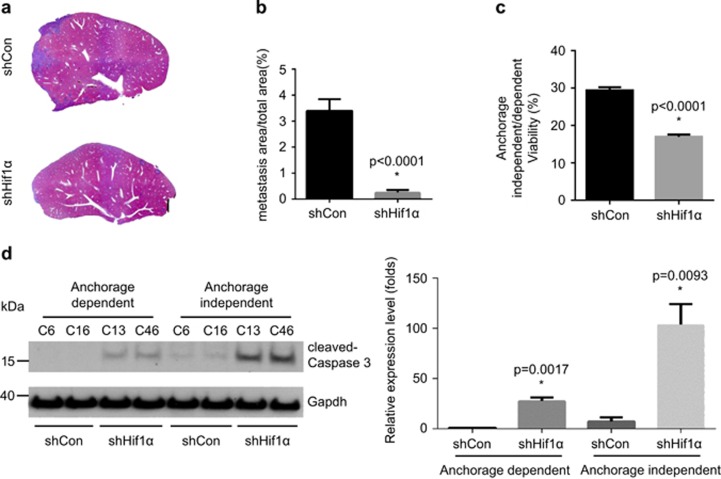
Loss of Hif1α impairs hepatic metastasis and resistance toward anoikis state. (**a–b**) Representative H&E staining pictures (**a**) and metastasis area calculation (**b**) reveal decreased metastatic foci after portal vein injection of shHif1α cells as compared with shControl cells. (**c**) Anoikis assay shows diminished cell survival in the shHif1α cells under anchorage-independent conditions. (**d**) Western blot and quantified measurements show expression of cleaved-caspase 3 in shControl or shHif1α cells under anchorage-dependent and -independent conditions. The data were a result of three independent experiment. An unpaired *t*-test was used for determining statistical significance. *P<0.05. See Supplementary Materials and Methods.

To test the function of Hif1α in tumour colonization/metastasis, we inoculated shControl and shHif1α cells into the portal vein. Here, shHif1α cells developed significantly less hepatic metastasis than control cells (Figure 4a). The percentage of the area of metastasis in the liver (including median, left, right and caudate lobe) of shHif1α cells-injected animals was 13.3-fold lower (*P*<0.0001) than that of control cells (Figure 4b). These data show that the ability of tumour colonization was dramatically decreased after Hif1α knock-down. Since being able to survive under anchorage-independent conditions is an initial step of tumour colonization, we performed an anoikis assay to test this. In line, the viability of shHif1α cells under anchorage-independent conditions was significantly reduced in comparison with control cells (Figure 4c, 43% reduction in viability, *P*<0.0001). In accordance with above described data, PDAC cells became more susceptible to anchorage-independent induced cell deaths after Hif1α knock-down (Figure 4d, 14.0 fold change, *P*=0.0093).

In summary, we tested the *in vivo* function of Hif1α in two major tumour-associated biological scenarios: primary tumour growth and remote colonization. Notably, although Hif1α depletion generally renders PDAC cells more susceptible to stress-induced (that is, hypoxia or loss of cell/ECM contact) cell deaths, which is in line with the general function Hif1α, it leads to different oncogenic consequences. In particular, the results of our study partially support previous data showing that genetic ablation of Hif1α significantly accelerated oncogenic Kras-driven pancreatic tumorigenesis potentially by activating B cells-mediated inflammation. However, no difference was observed in B-cell infiltration in the current study. This disparity could be attributed to the different mouse models (xenograft vs genetic models). The used xenograft model lacks the co-evolution of PDAC cells and the immune system. In the genetic model, Hif1α is concomitantly inactivated in the endocrine cells (for example, β cells), which is known to cause insulin resistance,^12^ which may influene the subsequent immune response. Despite these limitations, two studies led to the similar conclusion that Hif1α is crucial for tumour growth and in modulating the immunogenic reactions towards PDAC.

Considering this context-dependent role Hif1α in pancreatic tumorigenesis, clinical trials of Hif1α inhibitors (for example, PX-478) in PDAC need to be carried out with caution.^13, 14^ Based on the evident crosstalk between Hif1α and the immune system, Hif1α inhibitors could be tested in combination with various immune therapies in PDAC (for example, PD-1 inhibitors^15^). On the other hand, since Hif1α dramatically affects the capacity of PDAC in tumour colonization/metastasis, Hif1α inhibitors might be effective in targeting circulating cancer cells. In this regard, both genetic and xenograft models are useful in testing the effectiveness of such therapies.

## References

[bib1] Kong B, Cheng T, Wu W, Regel I, Raulefs S, Friess H et al. Hypoxia-induced endoplasmic reticulum stress characterizes a necrotic phenotype of pancreatic cancer. Oncotarget 2015; 31: 32154–32160. 10.18632/oncotarget.5168 PMC4741665 26452217

[bib2] Koong AC, Mehta VK, Le QT, Fisher GA, Terris DJ, Brown JM et al. Pancreatic tumors show high levels of hypoxia. Int J Radiat Oncol Biol Phys 2000; 48: 919–922. 11072146 10.1016/s0360-3016(00)00803-8

[bib3] Semenza GL. Regulation of mammalian O_2_ homeostasis by hypoxia-inducible factor 1. Annu Rev Cell Dev Biol 1999; 15: 551–578. 10611972 10.1146/annurev.cellbio.15.1.551

[bib4] Kumar H, Choi DK. Hypoxia inducible factor pathway and physiological adaptation: a cell survival pathway? Mediators Inflamm 2015; 2015: 584758. 26491231 10.1155/2015/584758 PMC4600544

[bib5] Colbert LE, Fisher SB, Balci S, Saka B, Chen Z, Kim S et al. High nuclear hypoxia-inducible factor 1 alpha expression is a predictor of distant recurrence in patients with resected pancreatic adenocarcinoma. Int J Radiat Oncol Biol Phys 2015; 91: 631–639. 25596110 10.1016/j.ijrobp.2014.11.004 PMC5746186

[bib6] Ye LY, Zhang Q, Bai XL, Pankaj P, Hu QD, Liang TB. Hypoxia-inducible factor 1alpha expression and its clinical significance in pancreatic cancer: a meta-analysis. Pancreatology 2014; 14: 391–397. 25278309 10.1016/j.pan.2014.06.008

[bib7] Shibaji T, Nagao M, Ikeda N, Kanehiro H, Hisanaga M, Ko S et al. Prognostic significance of HIF-1 alpha overexpression in human pancreatic cancer. Anticancer Res 2003; 23: 4721–4727. 14981919

[bib8] Zhong H, Chiles K, Feldser D, Laughner E, Hanrahan C, Georgescu MM et al. Modulation of hypoxia-inducible factor 1alpha expression by the epidermal growth factor/phosphatidylinositol 3-kinase/PTEN/AKT/FRAP pathway in human prostate cancer cells: implications for tumor angiogenesis and therapeutics. Cancer Res 2000; 60: 1541–1545. 10749120

[bib9] Lee KE, Spata M, Bayne LJ, Buza EL, Durham AC, Allman D et al. Hif1a deletion reveals pro-neoplastic function of B cells in pancreatic neoplasia. Cancer Discov 2016; 6: 256–269. 26715642 10.1158/2159-8290.CD-15-0822 PMC4783189

[bib10] Kong B, Wu W, Cheng T, Schlitter AM, Qian C, Bruns P et al. A subset of metastatic pancreatic ductal adenocarcinomas depends quantitatively on oncogenic Kras/Mek/Erk-induced hyperactive mTOR signalling. Gut 2016; 65: 647–657. 25601637 10.1136/gutjnl-2014-307616

[bib11] Mantovani A, Allavena P, Sica A, Balkwill F. Cancer-related inflammation. Nature 2008; 454: 436–444. 18650914 10.1038/nature07205

[bib12] Cheng K, Ho K, Stokes R, Scott C, Lau SM, Hawthorne WJ et al. Hypoxia-inducible factor-1alpha regulates beta cell function in mouse and human islets. J Clin Invest 2010; 120: 2171–2183. 20440072 10.1172/JCI35846 PMC2877560

[bib13] Koh MY, Spivak-Kroizman T, Venturini S, Welsh S, Williams RR, Kirkpatrick DL et al. Molecular mechanisms for the activity of PX-478, an antitumor inhibitor of the hypoxia-inducible factor-1alpha. Mol Cancer Ther 2008; 7: 90–100. 18202012 10.1158/1535-7163.MCT-07-0463

[bib14] Zhao T, Ren H, Jia L, Chen J, Xin W, Yan F et al. Inhibition of HIF-1α by PX-478 enhances the anti-tumor effect of gemcitabine by inducing immunogenic cell death in pancreatic ductal adenocarcinoma. Oncotarget 2015; 6: 2250–2262. 25544770 10.18632/oncotarget.2948 PMC4385849

[bib15] Noman MZ, Desantis G, Janji B, Hasmim M, Karray S, Dessen P et al. PD-L1 is a novel direct target of HIF-1alpha, and its blockade under hypoxia enhanced MDSC-mediated T cell activation. J Exp Med 2014; 211: 781–790. 24778419 10.1084/jem.20131916 PMC4010891

