# Supplementary material for: In vivo functional dissection of a context-dependent role for Hif1α in pancreatic tumorigenesis
Source: Oncogenesis. 2016 Dec 12;5(12):e278–. doi: 10.1038/oncsis.2016.78 (PMC5177776; doi:10.1038/oncsis.2016.78)
Supplement: Supplementary Methods [file oncsis201678x1.docx]

**Supplemental Materials and Methods**

**Cell culture and stable transfection experiment**

The murine PDAC cell line was generated from the tumour developed in a genetically engineered mouse genotyping p48^Cre/+^; LSL-Kras^G12D/+^; Tsc1^fl/+^ as described before^1^. The cells were cultured in DMEM high glucose medium (D5796, Sigma-Aldrich, Munich, Germany) supplemented with 10% FBS, 100 u/ml of penicillin, 100 µg/ml of streptomycin at 37℃, 5% CO2.

For the generation of stable transfected clones, murine PDAC cells were transfected with 1 µg of either Hif1α shRNA plasmid (sc-35562, Santa Cruz Biotechnology, Heidelberg, Germany) or control shRNA plasmid (sc-108060, Santa Cruz Biotechnology, Heidelberg, Germany). Single clones were selected by culture medium supplemented with 2 µg/ml puromycin (sc-108071, Santa Cruz Biotechnology, Heidelberg, Germany).

**Western blot**

Nuclear proteins of shControl or shHif1α cells were extracted by using NE-PER™ Nuclear and Cytoplasmic Extraction Reagents (78833, Thermo Fisher Scientific, Dreieich, Germany). Total protein was prepared with diluted cell lysis buffer (9803, Cell Signalling Technology, Frankfurt am Main, Germany) supplemented with protease inhibitor (04693159001, Roche, Penzburg, Germany) and phosphatase inhibitor (04906837001, Roche, Penzburg, Germany). The expression of Hif1α, cleaved-caspase 3 was examined by using the anti-Hif1α antibody (NB100-105, Novus, Abingdon, UK) and the anti-cleaved-Caspase 3 antibody (9661, Cell Signalling Technology, Frankfurt am Main, Germany). Gapdh (sc-25778, Santa Cruz Biotechnology, Heidelberg, Germany) was used as housekeeping gene.

**Cell metabolic state assay**

The same number of cells in shControl or shHif1α group were cultured for 24 hours before the experiment. Cellular glucose uptake was determined according to a previously published protocol^2^. Intracellular glutamate levels were determined by using glutamate assay kit (ab83389, Abcam, Cambridge, UK). The secretion of lactate was measured with Cobas 8000 modular analyzer series (Roche, Penzburg, Germany) at the clinical chemistry department of Klinikum Rechts der Isar (TUM, Munich, Germany).

**Vegfa ELISA experiment**

The same number of shcontrol or shHif1α cells were seeded in a 6-well plate. After adherence, cells were incubated with 1 ml of FBS-free culture medium for 24 hours before the supernatant was collected. The Vegfa secretion in each group was determined by using a commercial mouse VEGF quantikine ELISA Kit (MMV00, R&D Systems, Wiesbaden, Germany).

**Colony formation**

3×10^2^ of shControl or shHif1α cells were seeded in 6 -well plates and continuously cultured for 7 days. Afterwards, 100% methanol was used for fixation and 10% of crystal violet was applied for colour staining. The colony number (containing more than 50 cells) was counted by two independent researchers.

**Inflammatory indicators measurements**

The inflammatory levels in mouse serum were determined by measuring SAA (ab157723, Abcam, Cambridge, UK), Il6 (562236, BD Biosciences, Heidelberg, Germany) and TNFα (562336, BD Biosciences, Heidelberg, Germany) ELISA according to the companies´ instructions.

**Immunohistochemistry staining and positive cell calculation**

The tissue section was deparaffinised, rehydrated and antigen retrieval was performed with either citrate buffer (pH 6.0; 10 mM citric acid, 0.05% Tween 20) or proteinase K (S3020, Dako, Hamburg, Germany). After endogenous peroxidase and non-specific binding blocking, the sections were incubated with anti-cleaved-Caspase 3 (9664, Cell Signalling Technology, Frankfurt am Main, Germany); anti-phospho-Histone H3 (9701, Cell Signalling Technology, Frankfurt am Main, Germany); anti-myeloperoxidase (CMC28917023, Cell Marque, Rocklin, USA)); anti-B220 (MAB1217, R&D Systems, Wiesbaden, Germany); anti-CD45 (550539, BD Biosciences, Heidelberg, Germany), anti-F4/80 (MF48000, Thermo Fisher Scientific, Dreieich, Germany) and anti-CD3 (ab16669, Abcam, Cambridge, UK) antibodies. After rabbit HRP-labelled anti-rat antibody (P0450, Dako, Hamburg, Germany) or goat HRP-labelled polymer anti-rabbit antibody (K4003, Dako, Hamburg, Germany) incubation, colour reaction was performed with Liquid DAB+ Substrate Chromogen System (K3468, Dako, Hamburg, Germany). Then the sections were counterstaining with Mayer’s hematoxylin, dehydrated and mounted.

For calculation of positively stained cell, five random views of pictures were taken by a microscope (Carl Zeiss, Munich, Germany) under 20x (for pH-H3) or 40x (for all immune cell staining) objective lens for every section. The number of stained cells in every picture was counted by two independent researchers using the ImageJ software (NIH).

**Anoikis assay**

For anoikis assay, 5×10^4^ cells were seeded either in an anchorage resistant plate (07-200-602, Sigma-Aldrich, Munich, Germany) or a standard 24-well plate for 24 hours. Afterwards, 250 µg of MTT (M5655, Sigma-Aldrich, Munich, Germany) was applied to each plate and kept for 4 hours before adding 500 µL of cell lysis buffer (10% SDS, 0.01 M HCl). The absorbance value was determined at 570 nm and the survival rate was calculated accordingly.

**Tumour cell transplantation experiment and metastatic area calculation**

For orthotopic pancreatic tumour transplantation experiments, 1x10^6^ of shControl or shHif1α cells were transplanted into the pancreas of 8 weeks old wide-type mice. After 4 weeks, the operated mice were sacrificed, pancreatic tumours and serum were collected. Tumour volumes were measured and calculated ex vivo. The study was performed under the agreement of a protocol approved by the Animal Care and Use Committee of the Technical University of Munich (ethical application approval no. 42-13).

For hepatic metastasis experiment, 5x10^5^ of tumour cells were injected into the portal vein of wild type mice and kept for 2 weeks. Afterwards, the mice were sacrificed, and the whole liver was collected. H&E staining was performed on tissue sections to determine histological features. Afterwards, every slide was scanned with a 1.25× object by using Zeiss Axio microscope. The total metastatic burden was determined as a ratio of the cross-sectional area occupied by the tumour to the liver area using ImageJ (NIH).

**Statistical analysis**

The GraphPad Prism 6 (GraphPad) was used for statistical analysis. All experiments were repeated three times unless stated otherwise. All values are presented as mean ± standard error of the mean (SEM). For analysis, an unpaired Student’s t-test was used, p<0.05 was defined as the level of significance.

**Reference**

1 Kong B, Wu W, Cheng T, Schlitter AM, Qian C, Bruns P *et al*. A subset of metastatic pancreatic ductal adenocarcinomas depends quantitatively on oncogenic Kras/Mek/Erk-induced hyperactive mTOR signalling. Gut 2016; 65: 647-657.

2 Yamamoto N, Ueda M, Sato T, Kawasaki K, Sawada K, Kawabata K *et al*. Measurement of glucose uptake in cultured cells. Curr Protoc Pharmacol 2011; Chapter 12: Unit 12 14 11-22.
